# Characterization of the complete *Salix viminalis* var. *gmelinii* Turcz 1854 chloroplast genome from the northeast of China

**DOI:** 10.1080/23802359.2022.2127337

**Published:** 2022-10-06

**Authors:** Jie Wan, Huijie Tang, Zicheng Yu, Jing Wang, Xu Yao, Xiaoping Li

**Affiliations:** a Collaborative Innovation Center of Southern Modern Forestry, Nanjing Forestry University, Nanjing, China; b College of Forestry, Nanjing Forestry University, Nanjing, China; c Key Laboratory of Forest Tree Genetics and Breeding and High-Efficiency Cultivating in Jiangsu Province, Nanjing Forestry University, Nanjing, China

**Keywords:** Chloroplast genome, phylogenetic tree, Salicaceae, *Salix viminalis* var. *gmelinii*

## Abstract

The *Salix viminalis* var*. gmelinii* Turcz 1854 is a variant of the *Salix* genus, from the Salicaceae family, and possesses an extremely high economic value. In this study the complete chloroplast genome of the woody plant *S. viminalis* var. g*melinii* was characterized for the first time using a high-throughput approach in conjunction with *de novo* assembly technology. The *S.viminalis* var. *gmelinii* chloroplast genome is 155,405 base pairs (bp) in length and contains 36.71% GC content. It incorporates a large single-copy region (LSC, 84,287bp) alongside one small-copy region (SSC, 16,198bp), and two inverted repeat regions (IRA and IRB, 27,460bp). Moreover, this chloroplast genome encodes 128 genes, which comprises 83 protein-coding genes, 37 transfer RNA (tRNA) genes, and eight ribosomal RNA (rRNA) genes. Furthermore, the phylogenetic analysis revealed that *S.viminalis* var. *gmelinii* is closely related to *S. cupularis* and *S.gordejevii*.

*Salix viminalis* var. g*melinii* is an exuberant shrub species that belongs to the genus *Salix,* which is the largest genus of Salicaceae worldwide and contains approximately 500 species (Argus 1997). Mainly distributed in the provinces of Heilongjiang, Jilin, Liaoning, and the autonomous region of Inner Mongolia in the northeast of China, the *S. viminalis* var. *gmelinii* grows on the banks of rivers and streams as a revetment tree. As a species, it provides many economical purposes, whereby its branches are used to produce weaving baskets, the leaves act to feed silkworms, while tannins are extracted from the bark. However, the characteristics of the *Salix* genus vary significantly between species and populations, while the similar natural hybrid phenomenon blurs the morphological boundaries between the species (Chen et al. 2010). Moreover, the genomic and related molecular genetic resources are limited, which makes it difficult to analyze. This study herein provides the complete chloroplast genome sequence of the *S. viminalis* var. *gmelinii*. The publication of which will both enhance the ability to undertake the phylogenetic study of the Salicaceae family and provide essential genetic information for the further development of *S. viminalis* var. *gmelinii*.

Fresh and healthy leaves of *S.viminalis* var. *gmelinii* were collected from Mao’er Mountain (Heilongjiang, China; coordinates: 45°23′N, 127°32′E), and the voucher specimen was deposited in the Biotechnology Building, Nanjing Forestry University, China (Li Xiaoping1, xpli@njfu.edu.cn, DBNHL2017005). The genomic DNA of *S.viminalis* var. *gmelinii* was extracted using the CTAB method (Doyle and Doyle 1987). Whole genomic sequencing was performed by an Illumina NovaSeq 6000 series sequencer (PE150) (Illumina, SanDiego, CA) following the completion of the sequencing library. A total of 2.61 G raw data were generated and then trimmed using Fastp (Chen et al. 2018). The chloroplast genome of *S.viminalis* var. *gmelinii* was assembled *de novo* using NOVOPlasty (version 4.1) (https://github.com/ndierckx/NOVOPlasty) (Dierckxsens et al. 2017). Subsequently, the assembled chloroplast genome sequence was annotated using an online GeSeq program (Tillich et al. 2017) alongside CPGAVAS 2 (Shi et al. 2019; Zhou et al. 2021) *S.gordejevii* (NC_058001.1) was used as the reference genome and manual correction was performed. After manual correction, the result annotation of the complete chloroplast DNA was submitted to NCBI online by BankIt (GenBank accession number: OK505606.1).

*S.viminalis* var. *gmelinii* represents a typical quadripartite structure, It is 155,405 bp in length and contains a small single-copy region (SSC,16,198 bp), a large single-copy region (LSC,84,287 bp) and a pair of inverted repeat regions (IR,27,460 bp). The total GC content of the chloroplast genome is 36.71%. A total of 128 genes were annotated from the chloroplast genome sequence, including 83 protein-coding genes, 37 transfer RNA (tRNA) genes, and eight ribosomal RNA (rRNA) genes. Moreover, a total of 18 genes, including seven protein-coding genes, seven tRNAs, and four rRNAs were duplicated in the IR regions.

To reveal the phylogenetic position of *S. viminalis* var. *gmelinii* against the close relatives of Salicaceae, the phylogenetic analysis was performed based on 22 complete chloroplast genomes of Salicaceae (Figure 1). The complete chloroplast genomes were aligned using MAFFT v7.307 (https://mafft.cbrc.jp/alignment/software/) (Katoh and Standley 2013). A phylogenetic tree was generated through a maximum likelihood (ML) analysis using MEGA 6.0 with 1000 bootstrap replicates and setting the *Arabidopsis thaliana* to the outgroup (Tamura et al. 2013). Comparison of the *Salix viminalis* var. *gmelinii* plastome to previously published data shows a high level of gene synteny with some publicly available *Salix* sequences (Zhou et al. 2021). And, there are three *Salix viminalis* plastomes deposited in the GenBank. They were placed in the phylogenetic tree for analysis and found that they did not form a monophyletic clade. The potential reasons for this phenomenon may be that they did not come from the same ancestor, or due to different sampling locations and times. The finished phylogenetic tree indicated that the chloroplast genome of *S. viminalis* var. *gmelinii* exhibited a close relationship with the *S. cupularis* and the *S. gordejevii* (Figure 1). The complete chloroplast genome sequence together with the gene annotations was deposited in the GenBank under the accession number OK505606. The publication of the complete chloroplast genome of *S. viminalis* var. *gmelinii* will potentially provide the necessary genetic resources and background data that can enhance the evolution and application study of the Salicaceae.

**Figure 1.**
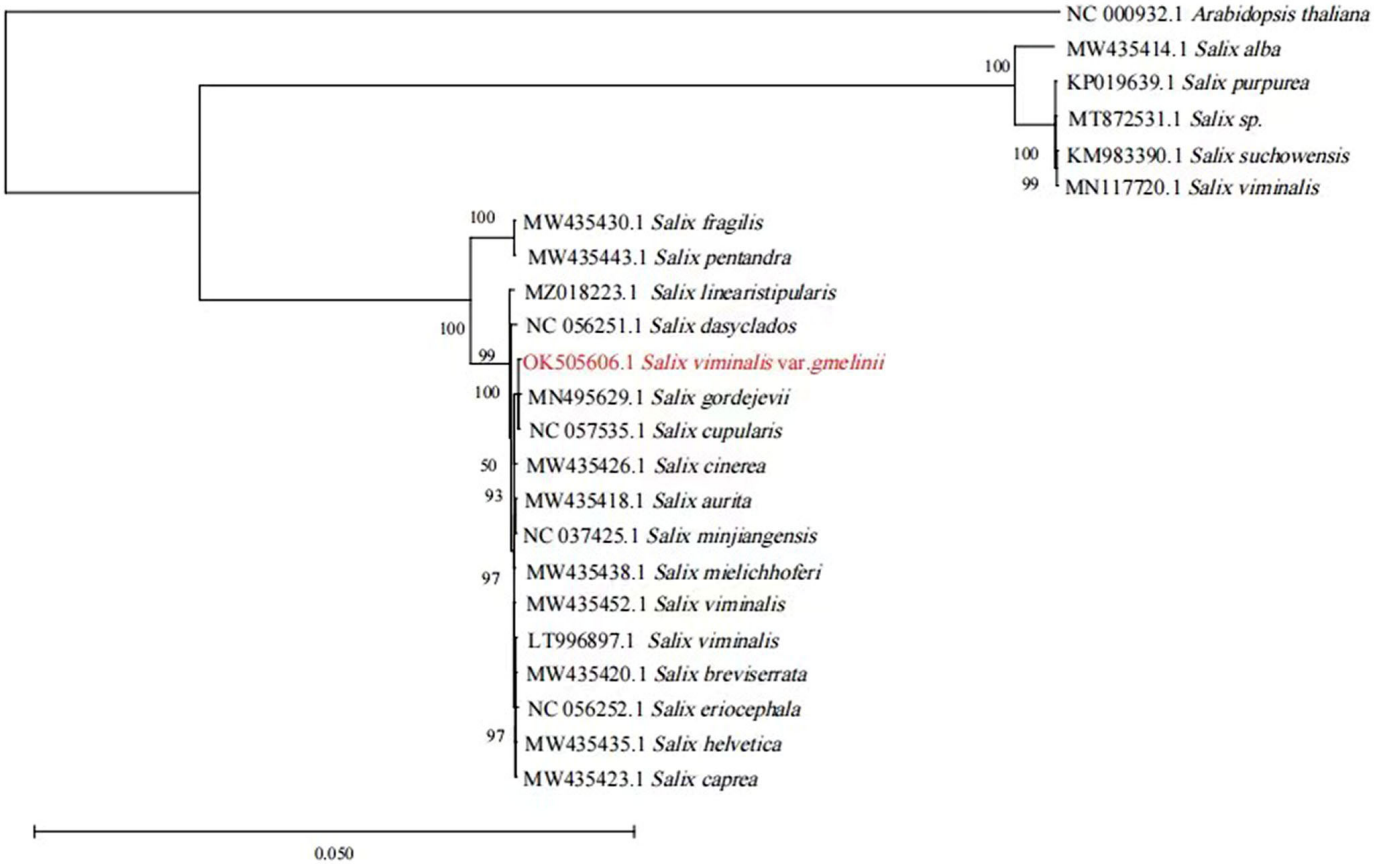
Maximum likelihood phylogenetic tree based on 22 selected plants chloroplast genome sequences.

## Data Availability

The complete chloroplast genome sequence was submitted to Genbank of NCBI (https://www.ncbi.nlm.nih.gov/nuccore/OK505606)under the reference number OK505606.The raw data has been deposited in SRA under accession number SRR16582939 (https://www.ncbi.nlm.nih.gov/sra/?term=PRJNA774245); The BioSample number and the BioProject number are SAMN22560793 PRJNA774245 respectively.

## Regulation statement

This study strictly follows the wild plant protection regulations of China, which were firstly promulgated by Decree No. 204 of the state council of China on Sept. 30, 1996 and amended on Oct.7, 2017 under Decision of The State Council on Revising Some Administrative Regulations.
